# Risk reduction strategies for *BRCA1/2* hereditary ovarian cancer syndromes: a clinical practice guideline

**DOI:** 10.1186/s13053-021-00196-9

**Published:** 2021-09-26

**Authors:** Michelle Jacobson, Nadia Coakley, Marcus Bernardini, Kelly-Ann Branco, Laurie Elit, Sarah Ferguson, Raymond Kim

**Affiliations:** 1 Women’s College Hospital, Toronto, ON Canada; 2 University of Toronto, Toronto, ON Canada; 3 Department of Oncology, McMaster University, Hamilton, ON Canada; 4 Ontario Health’s Cancer Care Ontario’s Program in Evidence-based care, McMaster University, Juravinski Site, G Wing, 2nd Floor room 227, 711 Concession Street, Hamilton, ON L8V 1C3 Canada; 5 Princess Margaret Cancer Center, University Health Network (UHN) Toronto, Mount Sinai, Toronto, ON Canada; 6 Patient Representative, Toronto, ON Canada; 7 Hamilton Health Sciences Centre-Juravinski Cancer Centre, Hamilton, ON Canada; 8 Ontario Health, Cancer Care Ontario, Toronto, ON Canada; 9 University Health Network (UHN), Toronto, ON Canada; 10 Princess Margaret Cancer Centre, Toronto, ON Canada; 11 Sick Kids Hospital, Toronto, ON Canada

**Keywords:** Cancer Care Ontario, Surgery, Systemic treatment, Ovarian cancer, BRCA1/2, HBOC, RRSO, Screening, Guideline recommendations

## Abstract

**Objective:**

The purpose of this guideline is to make recommendations regarding the care of women who harbour a pathogenic or likely pathogenic variant in *BRCA1* and *BRCA2*.

**Methods:**

Draft recommendations were formulated based on evidence obtained through a systematic review of RCTs, comparative retrospective studies and guideline endorsement. The draft recommendations underwent an internal review by clinical and methodology experts, and an external review by clinical practitioners.

**Results:**

The literature search yielded 1 guideline, 5 systematic reviews, and 15 studies that met the eligibility criteria.

**Conclusions:**

In women who harbour a pathogenic or likely pathogenic variant in *BRCA1* and *BRCA2 s*creening for ovarian cancer is not recommended*.* Risk-reducing surgery is recommended to reduce the risk of ovarian cancer. In the absence of contraindications, premenopausal women undergoing RRSO should be offered hormone therapy until menopause. Systemic hormone replacement therapy, is not recommended for women who have had a personal history of breast cancer. RRSO should be considered for breast cancer risk reduction in women younger than 50 years. After a breast cancer diagnosis, RRSO for breast cancer mortality reduction can be considered within two years to women who harbour a pathogenic or likely pathogenic variant in *BRCA1* if younger than the recommended age range for ovarian cancer risk reduction. RRSO before the age of 40 and specifically for breast cancer treatment in *BRCA*2 should be considered only if recommended by their breast cancer oncologist. Following RRSO, it is not recommended to do surveillance for peritoneal cancer.

## Introduction

In 2020, ovarian cancer will account for 4.9% of deaths from cancer in Canada [1]. Approximately 5 to 15% of these cancers will occur in women with the *BRCA1* and *BRCA2* genes [1]. In women with a hereditary ovarian cancer syndrome the cumulative chance of developing ovarian cancer to the age of 80 years is 44% for *BRCA1* and 17% for *BRCA2* carriers. This is significantly greater than the general population (1.7%) [2].

Many women at risk of ovarian cancer are recommended to undergo RRSO. However, this surgery causes infertility, premature menopause, and risks for early cardiovascular disease, cognitive decline, and osteoporosis if done before menopause [3]. Screening modalities described are mostly comprised of a CA125 blood test, and TVU. However, it is not known if these screening modalities actually help to detect cancer earlier or what the optimal timing should be for high-risk women. A viable ovarian cancer screening protocol is needed.

This systematic review has been registered on the PROSPERO website (International prospective register of systematic reviews) with the following registration number CRD42018110541. Details of the protocol for this systematic review were registered on PROSPERO and can be accessed at https://www.crd.york.ac.uk/prospero/display_record.php?RecordID=110541.

The Working Group members of the Risk Reduction for Hereditary Ovarian Cancer Syndromes GDG (Guideline Development Group) developed this evidentiary base to inform recommendations as part of a clinical practice guideline. Based on the objectives of this guideline, the Working Group derived the research questions outlined below.

1. In women who harbour a pathogenic or likely pathogenic variant in *BRCA1* and *BRCA2* and are at increased risk for epithelial ovarian, fallopian tube, or primary peritoneal cancer, does screening with either serial U/S, CA125 or ROCA (Risk of Ovarian Cancer Algorithm), decrease their risk of ovarian cancer?
2. In women who harbour a pathogenic or likely pathogenic variant in *BRCA1* and *BRCA2* and are at increased risk for epithelial ovarian, fallopian tube, or primary peritoneal cancer, what is the optimal strategy to prevent these cancers?
3. What is the optimal post-surgical management protocol to address the sequelae of RRSO (Risk-Reducing Salpingo-Oophorectomy) in women who harbour a pathogenic or likely pathogenic variant in *BRCA1* and *BRCA2*?

## Target population

These recommendations apply to women who harbour a pathogenic or likely pathogenic variant in *BRCA1* and *BRCA2*.

## Intended users

This guideline is targeted for: clinicians involved in the care of women who harbour a pathogenic or likely pathogenic variant in *BRCA1* and *BRCA2*.

## Development of recommendations

The Program in Evidence-based Care (PEBC) produces evidence-based and evidence-informed guidance documents using the methods of the Practice Guidelines Development Cycle [4, 5]. This process includes a systematic review, interpretation of the evidence by the Working Group and draft recommendations, internal review by content and methodology experts, patient and care giver review, and external review by Ontario clinicians and other stakeholders.

The project was led by a small Working Group of the Gynecologic GDG members, which was responsible for reviewing the evidence base, drafting the guideline recommendations, and responding to comments received during the document review process. The Working Group had expertise in surgical oncology, medical oncology, genetics and health research methodology.

## Literature search results

### Search for existing guidelines, systematic reviews and primary literature

As a first step in developing this guideline, a search for existing guidelines and systematic reviews was undertaken to determine if an existing guideline or systematic review could be adapted or endorsed. To this end, practice guideline databases, guideline developer websites along with Medline, the Cochrane Database of Systematic reviews and EMBASE (2004- July 242,020) were searched. Identified guidelines were evaluated using the AGREE II tool [6]. Any identified systematic reviews that addressed the research questions were assessed using A Measurement Tool to Assess Systematic Reviews (AMSTAR 2) [7]. The results of the AMSTAR 2 assessment were used to determine whether or not any existing review could be incorporated as part of the evidentiary base.

The search for guidelines and systematic reviews uncovered 6611 documents, of these, 119 underwent full-text review. One guideline, five systematic reviews and 15 studies from the primary literature were retained.

The Working Group members reviewed the guidelines in detail and reviewed each recommendation of that guideline to determine whether it could be endorsed, endorsed with changes, or rejected. This determination was based on the agreement of the Working Group members with the interpretation of available evidence presented in the guideline, and whether it was applicable and acceptable to the Ontario context, and whether new evidence since the guideline was developed might change any of the recommendations. When new evidence was available the recommendations were based on the new data.

### Study selection criteria and process

Included studies were published in English, examined serial U/S (Ultrasound), CA125, or ROCA in women to screen for ovarian cancer. Studies evaluating HRT (Hormone Replacement Therapy) in women who harbour a pathogenic or likely pathogenic variant in *BRCA1* and *BRCA2 were also included.* The minimum study size was 20 and participants had to have no prior oophorectomies. Studies were excluded if they were case studies, single arm studies, commentaries or editorials.

Data from the included guidelines, systematic reviews, and primary studies were extracted by one member of the Working Group (NC). The remaining authors reviewed the articles considered for inclusion and agreed on the full-text articles to be included. All extracted data and information were audited by an independent auditor (FM).

Important quality features, such as industry funding, control details, blinding, and power calculations, for each non-RCT (Randomized Clinical Trial) study were extracted. RCTs were evaluated using the Cochrane Risk of Bias tool [8].

## Recommendations, key evidence, and interpretation of evidence

**Table:** 

**Recommendation 1**	
Screening for ovarian, tubal, or primary peritoneal cancer is not recommended in women who harbour a pathogenic or likely pathogenic variant in *BRCA1* and *BRCA2*.	
**Qualifying Statements for Recommendation 1**	
• There is currently no screening method for ovarian, tubal, or primary peritoneal cancer that shows a survival benefit.	
• More data are required before any screening method for ovarian, tubal, and peritoneal cancer can be recommended.	
**Key Evidence for Recommendation 1**	
• Fifteen papers [3, 9–22] representing 13 individual studies were found.	
• The four randomized trials found no differences in survival with screening to detect ovarian cancer compared to usual care [14–16, 19].	
• Only two studies showed a slight benefit in survival [3, 13].	
• A stage shift was detected in the UK FOCSS study by Rosenthal et al. [3], but there is insufficient evidence that this screening method resulted in a survival benefit.	
**Justification for Recommendation 1**	
The Working Group members weighed the benefits and harms and determined that mortality was a key outcome. The evidence does not show a benefit for survival in screening for ovarian cancer.

**Table:** 

**Recommendation 2**	
Risk-reducing surgery is recommended to reduce the risk of ovarian cancer in women with a hereditary predisposition or risk. This is endorsed from Jacobson et al. 2018 [23].	
**Key Evidence for Recommendation 2**	
We endorse the recommendations from the clinical practice guideline conducted by Jacobson et al. [23] on behalf of the SOGC (The Society of Obstetricians and Gynaecologists of Canada). This guideline scored well on the AGREE II scale. The scores are reported in Table 4–3 in Section 4 of this document. The evidence underpinning the recommendations is comprised of one randomized study and one comparative study.	
**Justification for Recommendation 2**	
The Working Group members are confident in their endorsement of this recommendation. The source had adequate quality ratings, there is an excellent alignment with research questions of interest to the Working Group, methods and evidence and synthesis are convincing, and the treatments and patients included in the evidence base are generalizable to the Ontario context.

**Table:** 

**Recommendation 3**	
It is premature to recommend acetylsalicylic acid for ovarian cancer prophylaxis in women who harbour a pathogenic or likely pathogenic variant in *BRCA1* and *BRCA2*. This is endorsed from Jacobson et al. 2018 [23].	
**Qualifying Statements for Recommendation 3**	
• There is an ongoing clinical trial (NCT03480776) determining the effectiveness of the use of acetylsalicylic acid in ovarian cancer.	
**Key Evidence for Recommendation 3**	
We endorse the recommendations from the clinical practice guideline conducted by Jacobson et al. [23] on behalf of the SOGC. This guideline scored well on the AGREE II scale. The scores are reported in Table 4–3 in Section 4 of this document. The evidence underpinning the recommendations is comprised of 12 population-based case-control studies.	
**Justification for Recommendation 3**	
The Working Group members are confident in their endorsement of this recommendation. The source had adequate quality ratings, there is an excellent alignment with research questions of interest to the Working Group, methods and evidence and synthesis are convincing, and the treatments and patients included in the evidence base are generalizable to the Ontario context.

**Table:** 

**Recommendation 4**	
• • In the absence of contraindications, premenopausal women who harbour a pathogenic or likely pathogenic variant in *BRCA1* and *BRCA2* undergoing RRSO should be offered hormone therapy until the average age of menopause (age 51). • • Systemic HRT, at any age, is not recommended for women who harbour a pathogenic or likely pathogenic variant in *BRCA1* and *BRCA2* who have had a personal history of breast cancer. These women can be offered non-hormonal alternatives for vasomotor symptom management. • • Symptoms related to the genitourinary syndrome of menopause should be treated with moisturizers, lubricants, and local low-dose estrogen therapy as needed.	
**Qualifying Statements for Recommendation 4**	
• • The treatment of symptoms relating to the genitourinary syndrome of menopause in the third bullet point is based on accepted general practice and not *BRCA*-carrier-specific evidence.	
• • Where combination HRT is used, it is prudent to choose progesterone over synthetic progestins, or the TSEC (Tissue-Selective Estrogen Complex) [24].	
**Key Evidence for Recommendation 4**	
Five meta-analyses concerning HRT use in women who harbour a pathogenic or likely pathogenic variant in *BRCA1* and *BRCA2* were found [25–29]. The systematic review by Gordhandas et al. evaluated five studies that demonstrated that women who used HRT reported fewer endocrine symptoms (*p* < 0.05) and had similar levels of sexual functioning when compared to women without HRT after RRSO. Women had less discomfort (*p* = 0.001) and HRT reduced dyspareunia (*p* = 0.027) [26]. In the Gordhandas et al. systematic review bone health was assessed by three studies. The studies demonstrated that in women who used HRT the OR for bone disease was 1.2 (95% CI, 0.4 to 3.7). Another study showed that women who had been deprived of estrogen for greater than two years had a higher prevalence of bone loss compared with women who took HRT. Women who had not taken HRT after RRSO through at least age 45 had significantly higher mortality due to cardiovascular disease (HR, 1.84; 95% CI, 1.27 to 2.68, p = 0.001). Women who took HRT after RRSO had similar outcomes to women not undergoing RRSO (HR, 0.65; 95% CI, 0.30 to 1.41, *p* = 0.28) [26]. The risk of developing breast cancer was assessed by three systematic reviews. All three reviews showed that taking HRT was not associated with an increase in breast cancer diagnosis. The systematic review and meta-analysis by Marchetti et al. included three studies. The risk of breast cancer associated with HRT use after RRSO was 1.01 (95% CI, 0.16 to 1.54). When limited to prospective trials, the risk of breast cancer in women who harbour a pathogenic or likely pathogenic variant in *BRCA1* and *BRCA2* who used HRT did not have a negative impact (HR, 0.98; 95% CI, 0.63 to 1.52). A subgroup analysis on the type of HRT showed no significant difference in breast cancer risk for women who used estrogen alone compared to estrogen and progesterone. However, the breast cancer risk was lower for women who used estrogen alone versus estrogen and progesterone in the overall population (OR, 0.62; 95% CI, 0.29 to 1.31) [27]. The systematic review by Vermeulen et al. also examined the risk of breast cancer in women taking HRT following RRSO. Seven studies were evaluated and none of the studies showed that short-term use (2.8 to 4.3 years) was associated with an increase in breast cancer risk [29].

**Table:** 

**Recommendation 5**	
• RRSO should be offered to women who harbour a pathogenic or likely pathogenic variant in *BRCA1* after the age of 35 and *BRCA2* from between 40 and 45 years for ovarian/tubal/peritoneal carcinoma risk reduction.	
• For women diagnosed as pathogenic variant carriers after menopause, RRSO should be offered upon diagnosis.	
• RRSO should be considered for breast cancer risk reduction in women younger than 50 years who harbour a pathogenic or likely pathogenic variant in *BRCA2*.	
• After a breast cancer diagnosis, RRSO for breast cancer mortality reduction can be considered within two years to women who harbour a pathogenic or likely pathogenic variant in *BRCA1* if younger than the recommended age range for ovarian cancer risk reduction. RRSO before the age of 40 and specifically for breast cancer treatment in *BRCA*2 should be considered only if recommended by their breast cancer oncologist. This is endorsed from Jacobson et al. 2018 [23].	
**Qualifying Statements for Recommendation 5**	
• In a Canadian cohort study, 3722 unaffected women who harboured a pathogenic or likely pathogenic variant in *BRCA1* and *BRCA2* who had undergone only RRSO were followed until breast cancer diagnosis, prophylactic bilateral mastectomy, or death. In *BRCA1* carriers, HRs of breast cancer after RRSO were not significant at 0.96 (95% CI, 0.73 to 1.26), nor were they significant in *BRCA2* carriers (HR, 0.65; 95% CI, 0.37 to 1.16). However, when the latter group was stratified by age, RRSO had a significant reduction in breast cancer incidence when it was performed before the age of 50 years (HR, 0.18; 95% CI, 0.05 to 0.63) [30].	
**Key Evidence for Recommendation 5**	
We endorse the recommendations from the clinical practice guideline conducted by Jacobson et al. [23] on behalf of the SOGC. This guideline scored well on the AGREE II scale. The scores are reported in Table 4–3 in Section 4 of this document. The evidence underpinning the recommendations is primarily comprised of a guideline from 2017 and comparative studies.	
**Justification for Recommendation 5**	
The Working Group members are confident in their endorsement of this recommendation. The source had adequate quality ratings, there is an excellent alignment with research questions of interest to the Working Group, methods and evidence and synthesis are convincing, and the treatments and patients included in the evidence base are generalizable to the Ontario context.

**Table:** 

**Recommendation 6**	
• Bilateral salpingectomy alone for ovarian/tubal/peritoneal cancer risk reduction in women who harbour a pathogenic or likely pathogenic variant in *BRCA1* and *BRCA2* is still under investigation and should only be offered as an alternative to RRSO under a research protocol or if RRSO is an unacceptable choice for the patient.	
• Bilateral salpingectomy is an option for women who harbour a pathogenic or likely pathogenic variant in *BRCA1* and *BRCA2* who are younger than the recommended age for RRSO and do not wish to conceive further pregnancies (without assisted reproductive technologies).	
• The inclusion of hysterectomy with RRSO for harbour a pathogenic or likely pathogenic variant in *BRCA1* and *BRCA2* should be individualized, taking into account risk factors for uterine cancer, other uterine pathology, and tamoxifen use.	
• There are insufficient data to routinely recommend hysterectomy to reduce the risk of papillary serous uterine cancer in women who harbour a pathogenic or likely pathogenic variant in *BRCA1*.	
This is endorsed from Jacobson et al. 2018 [23]	
**Qualifying Statements for Recommendation 6**	
• A 2016 Dutch study examined mathematical models for ovarian cancer risk following two-step surgery in women who harbour a pathogenic or likely pathogenic variant in *BRCA1* and *BRCA2*. The investigators determined that whether salpingectomy offers (at its worst) a 35% risk reduction in ovarian cancer or (at its best) performs at the level of RRSO, an interval salpingectomy followed by bilateral oophorectomy five years later within the recommended window for preventive surgery affords risk reduction similar to that with RRSO alone [31].	
**Key Evidence for Recommendation 6**	
We endorse the recommendations from the clinical practice guideline conducted by Jacobson et al. [23] on behalf of the SOGC. This guideline scored well on the AGREE II scale. The scores are reported in Table 4–3 in Section 4 of this document. The evidence underpinning the recommendations is primarily comprised of a guideline from 2017 and comparative studies.	
**Justification for Recommendation 6**	
The Working Group members are confident in their endorsement of this recommendation. The source had adequate quality ratings, there is an excellent alignment with research questions of interest to the Working Group, methods and evidence and synthesis are convincing, and the treatments and patients included in the evidence base are generalizable to the Ontario context.

**Table:** 

**Recommendation 7**	
All RRSO for women who harbour a pathogenic or likely pathogenic variant in *BRCA1* and *BRCA2* should be performed by a skilled gynecologist. It is imperative that specimens be examined by an experienced pathologist familiar with the Sectioning and Extensively Examining the FIMbriated End technique and diagnostic criteria. Should an invasive or occult carcinoma be found, patients should be referred to a gynecologic oncologist. This is endorsed from Jacobson et al. 2018 [23].	
**Key Evidence for Recommendation 7**	
We endorse the recommendations from the clinical practice guideline conducted by Jacobson et al. [23] on behalf of the SOGC. This guideline scored well on the AGREE II scale. The scores are reported in Table 4–3 in Section 4 of this document. The evidence underpinning the recommendations is primarily comprised of comparative studies and one clinical practice guideline from 2015.	
**Justification for Recommendation 7**	
The Working Group members are confident in their endorsement of this recommendation. The source had adequate quality ratings, there is an excellent alignment with research questions of interest to the Working Group, methods and evidence and synthesis are convincing, and the treatments and patients included in the evidence base are generalizable to the Ontario context.

**Table:** 

**Recommendation 8**	
Post-oophorectomy care should be administered in an individualized manner, ensuring optimal QoL, bone health, and cardiovascular risk amelioration. This is endorsed from Jacobson et al. 2018 [23].	
**Qualifying Statements for Recommendation 8**	
• Because of the increased risk of osteoporosis following pre-mature menopause, undergoing dual x-ray absorptiometry scan one year following RRSO is suggested, then determining the future frequency based on those results.	
• Cardiovascular disease risk should be followed and ameliorated by the primary care practitioner or internist, while encouraging healthy lifestyle choices for these women.	
**Key Evidence for Recommendation 8**	
We endorse the recommendations from the clinical practice guideline conducted by Jacobson et al. [23] on behalf of the SOGC. This guideline scored well on the AGREE II scale. The scores are reported in Table 4–3 in Section 4 of this document. The evidence underpinning the recommendations is based on expert opinion.	
**Justification for Recommendation 8**	
The Working Group members are confident in their endorsement of the recommendation. The source had adequate quality ratings, there is an excellent alignment with research questions of interest to the Working Group, methods and evidence and synthesis are convincing, and the treatments and patients included in the evidence base are generalizable to the Ontario context.

**Table:** 

**Recommendation 9**	
Following RRSO, it is not recommended to do surveillance for peritoneal cancer in women who harbour a pathogenic or likely pathogenic variant in *BRCA1* and *BRCA2*. This is endorsed from Jacobson et al. 2018 [23].	
**Qualifying Statements for Recommendation 9**	
• Following the 90% risk reduction in ovarian/tubal cancer afforded by bilateral RRSO, the risk of peritoneal cancer is low (3.89% lifetime risk in *BRCA1*, 1.9% in *BRCA2*). No surveillance is recommended for women who have undergone RRSO [32–34].	
**Key Evidence for Recommendation 9**	
We endorse the recommendations from the clinical practice guideline conducted by Jacobson et al. [23] on behalf of the SOGC. This guideline scored well on the AGREE II scale. The scores are reported in Table 4–3 in Section 4 of this document. The evidence underpinning the recommendations is primarily comprised of comparative studies.	
**Justification for Recommendation 9**	
The Working Group members are confident in their endorsement of this recommendation. The source had adequate quality ratings, there is an excellent alignment with research questions of interest to the Working Group, methods and evidence and synthesis are convincing, and the treatments and patients included in the evidence base are generalizable to the Ontario context.	

## Related guidelines

Tinmouth J, Zwaal C, Gryfe R, Carroll JC, Baxter N, McCurdy BR, Ferguson SE. Cancer Screening for Persons at Risk for or Affected with Lynch Syndrome Evidence. Toronto (ON): Cancer Care Ontario; 2018 *October 22*. Program in Evidence-Based Care Guideline No.: 15-16es.

## Review and update

Guidelines developed by the PEBC are reviewed and updated regularly. Please visit the CCO Web site (http://www.cancercare.on.ca) for the full evidence-based series report and subsequent updates.

## Data Availability

The datasets used and/or analysed during the current study are available from the corresponding author on reasonable request.
